# Challenges in global improvement of oral cancer outcomes: findings from rural Northern India

**DOI:** 10.1186/1617-9625-10-5

**Published:** 2012-04-12

**Authors:** Jyoti Dangi, Taru H Kinnunen, Athanasios I Zavras

**Affiliations:** 1 Dept. of Oral Health Policy and Epidemiology, Harvard School of Dental Medicine, Harvard, Boston, MA; 2 Dept. of Epidemiology, Harvard School of Public Health, Harvard, Boston, MA; 3 Dept. of Oral Epidemiology and Biostatistics, Columbia University College for Dental Medicine, Columbia, New York, NY

## Abstract

**Background:**

In India, 72% of the population resides in rural areas and 30-40% of cancers are found in the oral cavity. The majority of Haryana residents live in villages where inadequate medical facilities, no proper primary care infrastructure or cancer screening tools and high levels of illiteracy all contribute to poor oral cancer (OC) outcomes. In this challenging environment, the objective of this study was to assess the association between various risk factors for OC among referrals for suscipious lesions and to design and pilot test a collaborative community-based effort to identify suspicious lesions for OC.

**Methods:**

Setting: Community-based cross sectional OC screening.

Participants: With help from the Department of Health (DOH), Haryana and the local communities, we visited three villages and recruited 761 participants of ages 45-95 years. Participants received a visual oral cancer examination and were interviewed about their dental/medical history and personal habits. Pregnant women, children and males/females below 45 years old with history of OC were excluded.

Main outcome: Presence of a suspicious oral lesion.

**Results:**

Out of 761 participants, 42 (5.5%) were referred to a local dentist for follow-up of suspicious lesions. Males were referred more than females. The referral group had more bidi and hookah smokers than non smokers as compared to non referral group. The logistic regression analysis revealed that smoking bidi and hookah (OR = 3.06 and 4.42) were statistically significant predictors for suspicious lesions.

**Conclusions:**

Tobacco use of various forms in rural, northern India was found to be quite high and a main risk factor for suspicious lesions. The influence of both the DOH and community participation was crucial in motivating people to seek care for OC.

## Background

While many oral health prevention measures have been adopted worldwide, their implementation is largely unknown in small and rural areas of developing countries. There is also little known as how one can prevent the occurrence of oral cancer (OC) in geographic areas with high risk factors that are unique to the developing world and in an environment of low literacy and fragmented health systems. For example, in rural India having 80% of elderly population [1], smokeless tobacco use is high, knowledge about health is low, and access to health care is a challenge.

India has one of the highest rates of OC in the world, accounting for one-third of the total cancer burden; this figure continues to rise and accounts for 50-70% of total cancer mortality [2]. Many epidemiological studies conducted over the last three decades in America, Europe and Asia have provided strong evidence of an association between alcohol and tobacco, resulting in an increased risk of oral and pharyngeal tumours [3]. In addition to the above factors, India has a high prevalence of chewing tobacco mixtures [4].

OC satisfies the criteria of a suitable disease for screening. Early detection of oral premalignant lesions and early neoplastic changes may be the best and most cost-effective means to improve survival and quality of life for OC patients from all socioeconomic communities [5]. Visual examination of the oral cavity is a simple approach to detect asymptomatic OC and precancerous lesions. The main aim of this pilot study was not to diagnose or treat oral cancer per se, but rather to assess the association between various risk factors for OC among referrals for suspicious lesions and to evolve a suitable model to screen a large number of people who are at extra risk for OC with little access to medical facilities and also to define the role of an international collaboration.

## Methods

The project was conceptualized and designed at Harvard University in Boston, Massachusetts and was reviewed and approved by the Ethics Committee of the Medical School on June 30^th^, 2009 as protocol number M-17448-101. The lesson of this project was to create a design and method to implement public health programs locally with international collaboration for OC prevention.

### Participants & settings

The study was carried out in three villages of Haryana during the months of July and August 2009. A total of 761 patients of age group 45-95 years participated in the study. Pregnant women, children and subjects diagnosed with OC prior to entry into the study were excluded.

The DOH contacted each village's local administrative unit to organize space and make arrangements for screening in villages. They recruited an interpreter from the local village who convinced the villagers about the benefits of screening and motivated them to participate in the study. They also helped in arranging participation from students of the local dental school.

### Questionnaire and intervention

The questionnaire included questions regarding the demographics, personal habits and both medical and dental history. The visual tactile examination included the findings of suspicious and non-suspicious lesions. The interpreter and two local dentists helped in obtaining the consent form, filling out the questionnaire and performing visual tactile examination (VTE).

Prior to conducting the study, there were three video training sessions for the informed consent, questionnaire and VTE. For the VTE, we conducted a pre-test at the beginning of the first session and a post-test at the end of the last session. Although calibration of a minimum score of 80% was not scored by the team during the pre-test, it was achieved in the post-test by replication of the trainings. Any lesion which was red, painless, firm, indurated and had a history of being unresolved for more than 14 days in the mouth was considered a suspicious lesion. The subjects with these lesions were referred to the local dentist for further follow-up, including the biopsy and diagnosis of OC.

### Statistical analysis

The variables used in this study for chi square and regression analysis included sociodemographic characteristics (gender, age, education and religion) and personal habit behaviours (bidi, hookah smoking and tobacco chewing). Logistic analysis was conducted to determine the associations between the predictor variables related to referrals for the follow-up after adjusting for gender, age, education and religion variables. The findings were analysed with STATA 10.

## Results

### Response Rate

780 participants were enrolled in the study. 19 adults did not participate in the study. Data was collected from 761 participants (response rate: 97.5%).

### Referral population

42 (5.5%) participants comprising of 33 (78.57%) males and 9 (21.43%) females were referred to local dentist for follow-up of suspicious lesions with mean age of 47.6 years (SD = +0.89) (Table 1). The male to female ratio was 3.6 to 1, 30 (71.42%) were Hindus and 25 (59.52%) had education less than high school.

**Table 1:** Socio-demographic characteristics and personal habit behaviours in referral and non referral population

		Referral	Non Referral	
**Variables**	**n (%)**	**n (%)**	**n (%)**	**p value**

	761	42 (5.52)	719 (94.48)	

**Socio-demographic variables:**				

*Gender*				

Males	379	33 (8.71)	346 (91.29)	0.000*

Females	382	9 (2.36)	373 (97.64)	

*Age*				

45-55 (reference group)	523	30 (5.75)	493 (94.25)	0.624

56-65	148	6 (4.05)	142 (95.95)	

66-75	59	5 (8.47)	54 (91.53)	

76-95	31	1 (3.23)	30 (96.77)	

*Religion*				

Hindus	537	30 (5.59)	507 (94.41)	0.062

Sikhs	199	8 (4.02)	191 (95.98)	

Muslims (reference group)	25	4 (16.00)	21 (84.00)	

*Education*				

Less than high school	442	25 (5.66)	417 (94.34)	0.967

High school	194	10 (5.15)	184 (94.85)	

College graduates (reference group)	125	7 (5.60)	118 (94.40)	

**Personal Habits Variables:**				

*Smoking bidi*				

Smokers	235	24 (10.21)	211 (89.79)	0.000*

Non Smokers (reference group)	526	18 (3.42)	508 (96.58)	

*Smoking hookah*				

Smokers	163	22 (13.50)	141 (86.50)	0.000*

Non Smokers (reference group)	598	20 (3.34)	578 (96.66)	

*Chewing tobacco*				

Chewers	144	13 (9.03)	131 (90.97)	0.041*

Non Chewers (reference group)	617	29 (4.70)	588 (95.30)	

*p < .05- significant

In Table 1, the data on personal habit behaviours shows that the referral group had more bidi (57.14%) and hookah smokers (52.38%) than non smokers as compared to the non referral group. In contrast, both the groups had less tobacco chewers. These differences were statistically significant (p < .05). Also, males were significantly referred more than females (p < .05). There were no differences between the two groups related to age, religion and education.

In Table 2, logistic regression analysis showed that smoking bidi and hookah were the most significant risk factors associated with the referral group as compared to non referral group (p = .000 for bidi and p = .001 for hookah).

**Table 2:** Risk factors associated with referrals using logistic regression

Personal Habit Variables:			
	**Odds Ratio (OR)**	**Confidence Interval (CI)**	***p value ***

*Smoking bidi*			

Smokers	3.063	1.606- 5.84	0.001*

Non Smokers (reference group)	1		

*Smoking hookah*			

Smokers	4.42	2.32-8.41	0.000*

Non Smokers (reference group)	1		

*Chewing tobacco*			

Chewers	1.97	0.97- 3.98	0.059

Non Chewers (reference group)	1	-	-

*p < .05- significant

Odds ratios and 95% CIs for predictors of suspicious lesions among referrals after adjusting for gender, age, education level and religion.

## Discussion

The results from the study showed that males were referred more than females. The referral group had more bidi and hookah smokers as compared to non referral group. On the other hand, both groups had less tobacco chewers indicating that tobacco chewing was not a predominant form of tobacco consumption in the population. Furthermore, regression analysis revealed that smoking bidi and hookah were found to be statistically significant risk factors among referrals for the suspicious lesions.

In our study, 42 adults were referred to the local dentist, which seems lower than previously reported studies from India [6]. We expected to find - and confirmed - more suspicious lesions in males than in females, based on the difference of tobacco use between the two sexes [7]. This finding agrees with various studies where oral squamous cell carcinoma was found to be more frequent in males than in females [8]. Our study also showed that smoking bidi and hookah were associated with higher risks of suspicious lesions. The odds ratio of smoking hookah is highest, as compared to smoking bidi or chewing tobacco. Other studies have found consistent results, and this finding deserves further investigation [9].

Challenges: There was a lack of knowledge about oral cancer and risk factors, lack of access to the healthcare system, lack of reliable public health screening programs at the population level to diagnose oral cancer early, lack of systematic efforts to educate the population and to support efforts to discontinue habits, lack of follow up on precancerous lesions and challenges encountered in low resource countries (such as lack of new generation diagnostics, imaging devices, chemotherapies, etc.).

To improve oral cancer outcomes, one needs to educate the population on risk factors (primary prevention), have programs to discontinue risk factors (secondary prevention), have programs to diagnose the disease early and a good referral and treatment system to treat oral cancer once diagnosed. Efforts are needed to streamline the above interventions and to "package" them into a sustainable "program" designed for low resource countries.

This pilot study has some strengths and limitations.

Strengths: There were several challenges while conducting the study, such as motivating villagers for screening, consent forms, the language barrier, transportation and inadequate dental facilities. The DOH helped in solving these challenges by recruiting an interpreter, two dental students and organizing space in local dispensaries for screening. In fact, the help from DOH was instrumental not only in obtaining all necessary permits and approvals, but also in creating the "bridges" and facilitating the community ties with the three villages. The interpreter and two dental students helped in motivating and recruiting eligible villagers as well as making announcements by going door to door to ensure full participation. They also helped in obtaining the consent form, filling out the questionnaire and performing VTE.

Limitations: In particular, cross-sectional studies are not designed to establish causal relationships, and thus caution should be exercised in interpreting the reported odds ratio. However, our intent was to collaborate with local authorities and the community to establish the prevalence of suspicious lesions in the population and to identify those at high-risk. Since the information on the habits was self-reported, there is a potential for information and recall bias. However, the information was gathered via structured interviews and the use of a questionnaire by a single examiner, minimizing the possibility of any misclassification of the exposure. Misclassification of the outcome is another possible limitation. We used a clinical examination protocol to identify suspicious oral lesions, but had no immediate access to adequate dental facilities to adjudicate the histology of the lesions. Eventually, all 42 patients with a positive finding were referred to a local dentist for follow-up and possible biopsy, but the follow-up information is not available to us.

Conclusion: Among the three major forms of tobacco use, hookah and bidi smoking were found to be statistically significant risk factors for suspicious lesions among the referral population. The DOH and community participation played a crucial role in motivating people for screening of OC to reduce the burden in high risk population with minimal access to health care.

## Competing interests

The authors declare that they have no competing interests.

## Authors' contributions

JD carried out the concept and design of the study, participated in the data collection, analysis and drafted the manuscript. THK performed the statistical analysis and helped to draft the manuscript. AIZ participated in the study's design, coordination and helped to draft the manuscript. All authors read and approved the final manuscript.
